# Variants in the *DNAH11* gene responsible for primary ciliary dyskinesia or probably atypical primary ciliary dyskinesia presenting left-right asymmetry disorder

**DOI:** 10.1371/journal.pone.0348352

**Published:** 2026-05-08

**Authors:** Kai Zhao, Lamei Yuan, Ying Xiong, Hong Xia, Sheng Deng, Ming Chen, Yunjie Liao, Jiangang Wang, Hao Deng

**Affiliations:** 1 Health Management Center, The Third Xiangya Hospital, Central South University, Changsha, China; 2 Center for Experimental Medicine, The Third Xiangya Hospital, Central South University, Changsha, China; 3 Research Center of Medical Experimental Technology, The Third Xiangya Hospital, Central South University, Changsha, China; 4 Disease Genome Research Center, Central South University, Changsha, China; 5 Department of Emergency, The Third Xiangya Hospital, Central South University, Changsha, China; 6 Department of Pharmacy, Xiangya Hospital, Central South University, Changsha, China; 7 Department of Radiology, The Third Xiangya Hospital, Central South University, Changsha, China; Dhofar University College of Medicine, OMAN

## Abstract

Primary ciliary dyskinesia (PCD) is a rare multi-system cilia-related disorder, and approximately 50% of individuals with PCD exhibit left-right asymmetry disorder. The dynein axonemal heavy chain 11 gene (*DNAH11*) pathogenic variants are responsible for primary ciliary dyskinesia 7, with or without left-right asymmetry disorder. This study aimed to detect the pathogenic variants in three unrelated patients diagnosed with PCD or left-right asymmetry disorder based on the clinical and imaging examinations. Whole exome sequencing, Sanger sequencing, and comprehensive bioinformatics analyses were performed. Seven *DNAH11* heterozygous variants, which involved evolutionarily conserved residues and were predicted to exert deleterious effects, reduce protein stability, change protein conformation, and affect non-covalent residue’s interactions, were identified as potential pathogenic factors responsible for these patients, respectively. In patient 1, three variants in compound heterozygotes, c.[3541A > G];[4334G > A;12428T > C] (p.[(Ser1181Gly)];[(Arg1445Gln;Met4143Thr)]), were confirmed. In patient 2, two variants in potential compound heterozygotes, c.2912A > G(;)7980A > T (p.(Asp971Gly)(;)(Gln2660His)), were detected. In patient 3, two variants in compound heterozygotes, c.[845T > C];[11402C > G] (p.[(Met282Thr)];[(Pro3801Arg)]), were confirmed. The phenotypes observed in these patients are consistent with typical/probably atypical PCD or *DNAH11*-associated ciliopathy, although functional validation is needed to confirm variant pathogenicity. These findings expand the phenotypic spectrum of *DNAH11* variants and may facilitate more accurate genetic diagnosis and counseling.

## Introduction

As a rare genetic condition first identified in 1936, primary ciliary dyskinesia (PCD, OMIM 244400) results from abnormal ciliary motility and structural defects, causing multi-organ involvement [1]. The impaired ciliary motion prevents the effective clearance of secretions from the respiratory tract, leading to recurrent infections, eventually bronchiectasis and impaired lung function. Respiratory distress, chronic wet cough, otitis media, rhinitis, sinusitis, and reduced fertility are common manifestations. Hydrocephalus, retinitis pigmentosa, and cystic lesions in organs like the liver and kidney, are reported in rare cases [2]. Despite its pan-ethnic nature, the prevalence of PCD varies, which was reported to be approximately 0.007%−0.010% and 0.002%−0.005% in Europe and the United States, respectively [3]. In a genetic database analysis, the minimum worldwide prevalence was estimated to be about 0.013% based on the known PCD-causing variants [4]. Remarkably, factors like diverse phenotypic features may contribute to underdiagnosis or misdiagnosis, leading to the assumed rarity, underestimate, or unavailable accurate data.

Though PCD is not easy to be clinically detected especially in those with atypical manifestations, it can be diagnosed earlier in those with left-right (LR) asymmetry disorder, accounting for approximately 50% of individuals with PCD [1]. In human and most animal embryos, LR asymmetry is established by motile cilium located at the node (i.e., nodal monocilium), which generates and directs the flow of extracellular fluids towards the node, thereby transmitting signals determining organ laterality [5]. The LR axis determination is one of essential and highly conserved processes in development. Situs solitus (SS) describes the normal positioning of thoraco-abdominal organs along the LR axis. Ciliary defects can cause LR asymmetry disorders, a group of heterogeneous disorders, which are characterized by abnormal organ placement or orientation across the LR axis, such as situs inversus (SI) and situs ambiguus (SA) [6]. SI manifests with complete, mirror-image reversal of normal visceral organs, and has an estimated incidence of 1 per 6,000 to 8,000 newborns. SA, more commonly known as heterotaxy, is defined as an abnormal arrangement of thoraco-abdominal organs relative to each other and the LR axis, not including SI. It is estimated to occur in about 1 out of 10,000 live births worldwide. Still, heterotaxy may be defined as strict SA plus complex congenital heart diseases. SI can often be asymptomatic, while SA can be accompanied by complex cardiac malformations, accounting for about 3% of congenital heart problems [7].

The inheritance of PCD, a genetically heterogeneous disorder, predominantly occurs in an autosomal recessive fashion, while some individuals may exhibit X-linked or autosomal dominant modes of transmission [2]. The first causative gene was reported in 1999. To date, at least 60 genes are associated with various PCD phenotypes, most of which are related to ciliary assembly, structure, and function. Among these PCD genes, no less than 36 are linked to defects in LR asymmetry [8]. Genes encoding dynein axonemal heavy chains are associated with ciliogenesis and LR patterning, in which the dynein axonemal heavy chain 11 gene (*DNAH11*) variants account for no less than 22% of the PCD cases [9].

In this study, variants in the *DNAH11* gene (NG_012886.2, NM_001277115.2, NP_001264044.1), c.[3541A > G];[4334G > A;12428T > C] (p.[(Ser1181Gly)];[(Arg1445Gln;Met4143Thr)]), c.2912A > G(;)7980A > T (p.(Asp971Gly)(;)(Gln2660His)), and c.[845T > C];[11402C > G] (p.[(Met282Thr)];[(Pro3801Arg)]), were identified as potential disease-associated variants in three unrelated Han-Chinese patients with PCD or LR asymmetry disorder, respectively.

## Methods and materials

### Participants and clinical evaluations

From Central South China, three probands belonging to separate Han-Chinese families (patient 1, I:1, Fig 1A; patient 2, II:1, Fig 1B; patient 3, II:1, Fig 1C) were included, diagnosed with PCD or LR asymmetry disorder based on the clinical and imaging examinations. Written informed consent was signed by the patients before peripheral venous blood was collected. Two respiratory physicians evaluated the subjects who underwent routine physical and imaging examinations, including chest X-ray, ultrasonography, and computed tomography (CT). Buccal swabs were obtained from the available family members with written informed consent. This study was conducted in compliance with the Declaration of Helsinki and received ethical clearance from the Institutional Review Board of the Third Xiangya Hospital, Central South University, which was carried out between December 2018 and December 2025.

**Fig 1:**
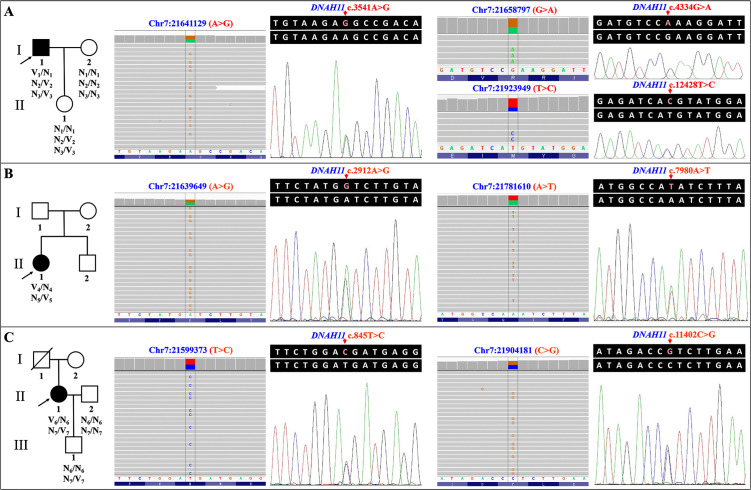
Pedigree figures, variants’ Integrative Genomics Viewer images, and Sanger sequencing results of the three patients from three unrelated Han-Chinese families with primary ciliary dyskinesia or left-right asymmetry disorder. **The arrow in the pedigree figure indicates the proband.** (A) The pedigree figure and genetic analysis results of patient 1. V_1_, the allele with the *DNAH11* c.3541A > G variant; V_2_, the allele with the *DNAH11* c.4334G > A variant; V_3_, the allele with the *DNAH11* c.12428T > C variant. (B) The pedigree figure and genetic analysis results of patient 2. V_4_, the allele with the *DNAH11* c.2912A > G variant; V_5_, the allele with the *DNAH11* c.7980A > T variant. (C) The pedigree figure and genetic analysis results of patient 3. V_6_, the allele with the *DNAH11* c.845T > C variant; V_7_, the allele with the *DNAH11* c.11402C > G variant. *DNAH11*, the dynein axonemal heavy chain 11 gene.

### Whole exome sequencing

Genomic DNA (gDNA) was extracted from peripheral venous blood utilizing a saturated phenol-chloroform extraction strategy previously reported (patient 1, 2, and 3) and buccal swabs using the HiPure Universal DNA Kit (Magen Biotech, Guangzhou, China) following the manufacturer’s instructions (offspring of patient 1 and 3) [10]. Whole exome sequencing (WES) of gDNA from three patients was performed by BGI-Shenzhen (Shenzhen, China). One microgram of gDNA specimen was randomly sheared into fragments by the Covaris sonication and fragments of 150–250 bp in size were selected. Selected fragments were both end-repaired, 3’-end “A” base added, and adaptor-ligated, and the processed DNA was further amplified by ligation-mediated PCR. The products were purified and further hybridized to the SureSelect^XT^ Human All Exon V6 kit (Agilent Technologies Inc., Santa Clara, CA, USA) for exome capture and enrichment, in which about 99% of the human exonic regions were covered via the biotin-labeled probes, firmly bound by the streptavidin-coated magnetic beads. The exome library was obtained from captured and purified exome fragments via amplification, denaturation, circularization, and digestion, and qualified DNA nanoballs were formed by rolling circle amplification. The BGISEQ-500 platform (BGI-Shenzhen) was utilized for high-throughput sequencing, as per the manufacturer’s guidelines.

### Variant analysis and validation

Sequencing raw data were filtered to generate clean data, which were subsequently mapped to the GRCh37/hg19 reference genome with Burrows-Wheeler Aligner (version 0.7.15). Picard tools (version 2.5.0) were employed to mark duplicate reads. The results of local alignment and base quality recalibration were obtained from Genome Analysis Toolkit (GATK). Sequence variants, including credible single nucleotide polymorphisms (SNPs) and insertions-deletions (indels), were detected by GATK HaplotypeCaller. Based on gene-level analysis and supported by provided databases, SnpEff software was employed for variant annotation and functional prediction. Variant prioritization was performed following the filtering steps as previously described [11–13]. Variants were filtered by some public variant databases including Single Nucleotide Polymorphism database (dbSNP, build 156), 1000 Genomes Project, the Exome Aggregation Consortium, and Genome Aggregation Database. To compensate for the small number of variant-positive cases, control databases were incorporated to better evaluate the background variant frequency and to interpret the rarity and potential clinical relevance of the identified variants. The in-house exome databases including 1,943 Chinese controls from BGI in-house exome database and 876 Chinese controls from our database were applied. Variants with the minor allele frequency lower than 0.01 were retained, in which coding SNPs, indels, and canonical splice-site alterations were prioritized. Databases like Online Mendelian Inheritance in Man (OMIM), ClinVar, and Human Gene Mutation Database, combined with the pathogenicity prediction tools, were applied to determine the potential disease-associated variants. Suspected deleterious variants in genes related to clinical phenotypes deposited in OMIM, as well as Human Phenotype Ontology (HPO), were noted, following the supposed inheritance pattern. Based on OMIM, 47 HPO annotations to the disease (ORPHA: 244) were revealed. Along with the clinical symptoms of three patients, nine terms were selected, including chronic rhinitis (HP: 0002257), nasal congestion (HP: 0001742), chronic sinusitis (HP: 0011109), hearing impairment (HP: 0000365), chronic otitis media (HP: 0000389), situs inversus totalis (HP: 0001696), productive cough (HP: 0031245), respiratory tract infection (HP: 0011947), and bronchiectasis (HP: 0002110). Next, Phen2Gene (https://phen2gene.wglab.org/) was applied for the prioritized gene list [14]. Further combining WES data and annotation results, the disease-associated gene variants for priority were determined. Prioritized variants as candidates in read alignments were further visualized using Integrative Genomics Viewer (version 2.19.6) by manual inspection [15], and final candidate variants were further tested by Sanger sequencing on an ABI 3730XL sequencer (Applied Biosystems Inc., Foster City, CA, USA) in the enrolled patients and available relatives. Primer3 (http://primer3.ut.ee/) was used to design the primer sequences, which are included in Table 1.

**Table 1:** The primers used for *DNAH11* variant identification.

Variant	Primer sequence	Product length (bp)
**c.845T > C**	5’-CACCGTCAAACGAAAGGATAA-3’	245
	5’-TTGCAGCTCAGGACACTTTG-3’	
**c.2912A > G**	5’-CATTGTGGTGGAAGGCTTTT-3’	246
	5’-TCCAGGTGTGTTGCTATTCG-3’	
**c.3541A > G**	5’-GGAGACAGATTCCGGACTTCA-3’	160
	5’-GCCATAGCTTTCCAAGAGGGT-3’	
**c.4334G > A**	5’-CCCCCAATCAGGTCAAGTTT-3’	229
	5’-CTCCCAGGACATCAAAAGGA-3’	
**c.7980A > T**	5’-CCTGGATACAGGGAGGAGTG-3’	244
	5’-TGCTATTGTTGCCTGGATCA-3’	
**c.11402C > G**	5’-TTCCCAGCAGGTATGGTTCT-3’	216
	5’-AAGTTAGGAAGTCAACGGGACTC-3’	
**c.12428T > C**	5’-GCAACAAAGCCAGACCTCAT-3’	544
	5’-ACCCGAGATTCCGCTTTTCT-3’	

*DNAH11*, the dynein axonemal heavy chain 11 gene.

### Bioinformatics analyses

The conservation of amino acid sequences among nine species was analyzed using the NCBI Basic Local Alignment Search Tool. Pathogenicity prediction online tools, including Sorting Intolerant from Tolerant (http://sift.jcvi.org/), Protein Variation Effect Analyzer (http://provean.jcvi.org/index.php), Polymorphism Phenotyping version 2 (http://genetics.bwh.harvard.edu/pph2/), and Combined Annotation Dependent Depletion (GRCh37-v1.7, https://cadd.gs.washington.edu/), were used to assess potential pathogenic effects of candidate variants. Protein structural changes introduced by the amino acid substitutions were predicted by Missense3D-DB (http://missense3d.bc.ic.ac.uk:8080/) [16]. Effects of substitutions on protein stability were predicted by MUpro (http://mupro.proteomics.ics.uci.edu/) using sequence information [17]. Differences among three groups (benign variants, reported pathogenic variants, and candidate variants) were further analyzed. The wild-type and variant-type protein structures were modeled using the online homology-modeling server SWISS-MODEL (https://swissmodel.expasy.org/) matched to the best template (8j07.908.A) in a monomeric state [18]. The Global Model Quality Estimation score for the predicted model was 0.68 and the target sequence coverage value was 0.99. The modeled structures were further analyzed by Visual Molecular Dynamics (version 1.9.3) [19]. The graphical representation of protein structure was drawn as a “NewCartoon” style with the coloring scheme of secondary structure (helix-sheet-turn-coil), in which the corresponding residue at the mutated site was highlighted in a selected color (magenta) and the zoomed-in view was further shown as a ball-and-stick (CPK) style. Non-covalent interactions at the atomic level in protein structures were explored by the online tool Residue Interaction Network Generator (https://ring.biocomputingup.it) [20]. The identified variants were further evaluated by the American College of Medical Genetics and Genomics-Association for Molecular Pathology (ACMG-AMP) guidelines of variant interpretation [21].

## Results

### Clinical findings

**Fig 2:**
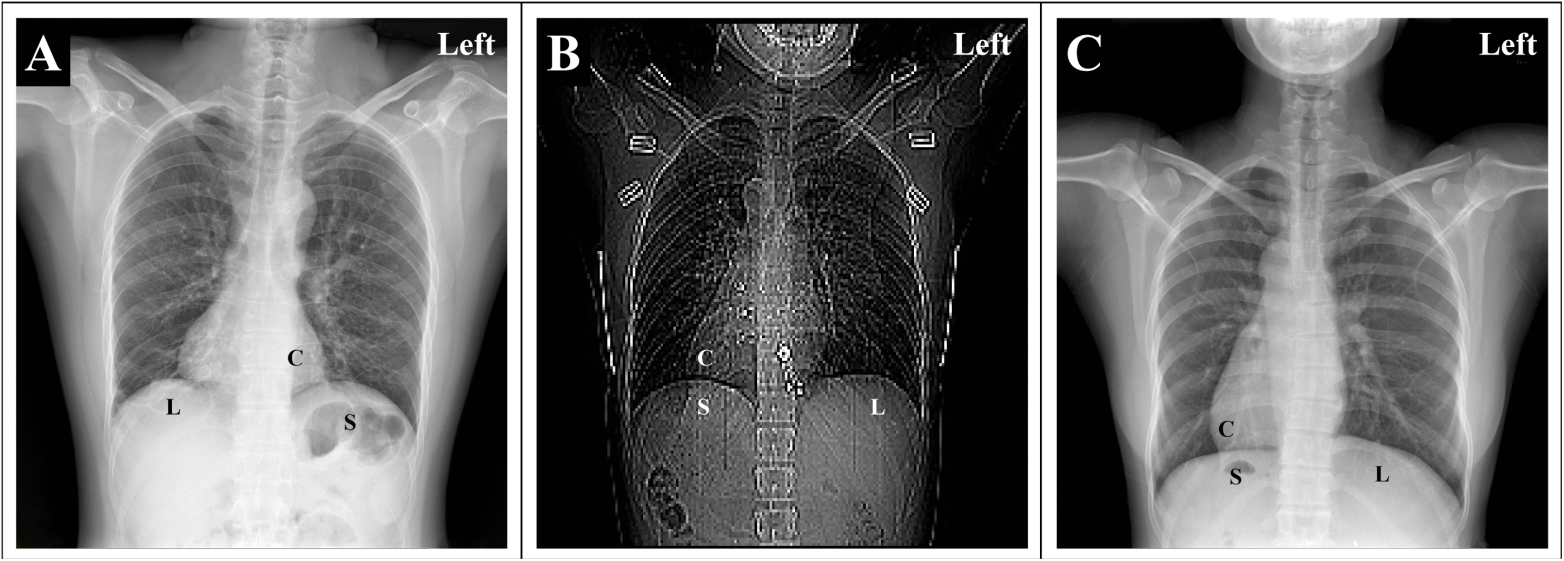
Radiology imaging of the three patients. (A) Chest X-ray of the patient 1 diagnosed with situs solitus revealed dextroposition, left stomach bubble, and right-sided liver. (B) Computed tomography of the patient 2 diagnosed with situs inversus revealed right cardiac apex, right stomach bubble, and left-sided liver. (C) Chest X-ray of the patient 3 diagnosed with situs inversus revealed right cardiac apex, right stomach bubble, and left-sided liver. C, cardiac apex; S, stomach bubble; L, liver.

The probands, three unrelated Han-Chinese patients, presented with PCD or LR asymmetry disorder. Patient 1, a 33-year-old man, was revealed to have a cardiac rightward displacement with a normal cardiac orientation (i.e., cardiac dextroposition) and the normal placement of the stomach bubble by chest radiograph (Fig 2A). Neither cardiac anatomical anomaly nor abdominal organ displacement was observed by cardiac or abdominal ultrasonography. Symptoms including chronic rhinitis and frequent pneumonia presented in early childhood, as well as the consequent sinusitis and bronchiectasis, and impaired hearing relating to chronic otitis media with recurrent infections was reported. Bronchial dilation test was negative. The clinical diagnosis was PCD. Patient 2 was a 26-year-old female diagnosed with SI and secondary pulmonary tuberculosis by CT scan (Fig 2B). Patient 3, a 42-year-old woman, was diagnosed with SI based on the mirror-image reversal of normal visceral organ asymmetry by chest radiograph (Fig 2C) and ultrasonic imaging. Patient 2 and 3 denied PCD-related symptoms like bronchiectasis, respiratory distress, and chronic oto-sino-pulmonary disorder. All three patients denied consanguinity and other comorbidities, and their relatives reported normal.

### Molecular genetic analysis

The WES revealed that patient 1 generated 14,409.15 Mb raw data, patient 2 produced 22,973.58 Mb raw data, and patient 3 produced 21,049.24 Mb raw data. After filtering from raw data, an average of 99.61% of clean reads aligned to the human reference genome (GRCh37/hg19) were obtained in three patients. For enough accuracy, the average target region sequencing depth was 126.02× in patient 1, 256.19× in patient 2, and 226.84× in patient 3. The total SNPs and indels detected in patient 1 were 130,652 and 21,566, respectively. The SNPs and indels detected in patient 2 were 105,112 and 18,344, respectively. A total of 103,206 SNPs and 17,730 indels were identified in patient 3.

Following the variant prioritization scheme, only seven heterozygous missense variants in the *DNAH11* gene, which is a known PCD gene and included in the seed gene list by Phen2Gene (score: 0.63282), were suspected as candidates for three unrelated patients. Three variants (c.3541A > G, p.(Ser1181Gly); c.4334G > A, p.(Arg1445Gln); c.12428T > C, p.(Met4143Thr)) in patient 1, two variants (c.2912A > G, p.(Asp971Gly); c.7980A > T, p.(Gln2660His)) in patient 2, and two variants (c.845T > C, p.(Met282Thr); c.11402C > G, p.(Pro3801Arg)) in patient 3 were identified (Fig 1). No other potential disease-associated variants in known phenotype-related genes were revealed. These seven variants are recorded in dbSNP156, with a low frequency in the general population and the BGI in-house exome database, but absent in our in-house exome database including 876 Chinese controls (Table 2). Sanger sequencing confirmed these heterozygous *DNAH11* variants in three patients, and revealed the presence or absence in the family members (Fig 1). In patient 1, the two variants, c.4334G > A and c.12428T > C, were transmitted to his daughter, whereas c.3541A > G was not, supporting c.3541A > G and c.4334G > A or c.12428T > C in the compound heterozygous state. In patient 3, the c.11402C > G variant was transmitted to her son, whereas c.845T > C was not, supporting the compound heterozygous state.

**Table 2:** Analysis of the *DNAH11* gene variants identified in three unrelated patients.

Items	Variant 1	Variant 2	Variant 3	Variant 4	Variant 5	Variant 6	Variant 7
**Chromosomal position (GRCh37/hg19)**	Chr7:21641129	Chr7:21658797	Chr7:21923949	Chr7:21639649	Chr7:21781610	Chr7:21599373	Chr7:21904181
**Chromosomal position (GRCh38/hg38)** ^a^	Chr7:21601511	Chr7:21619179	Chr7:21884331	Chr7:21600031	Chr7:21741992	Chr7:21559755	Chr7:21864563
**Exon**	18	24	76	15	49	4	70
**Nucleotide change (NM_001277115.2)**	c.3541A > G	c.4334G > A	c.12428T > C	c.2912A > G	c.7980A > T	c.845T > C	c.11402C > G
**Amino acid change (NP_001264044.1)**	p.(Ser1181Gly)	p.(Arg1445Gln)	p.(Met4143Thr)	p.(Asp971Gly)	p.(Gln2660His)	p.(Met282Thr)	p.(Pro3801Arg)
**Zygosity**	Heterozygote	Heterozygote	Heterozygote	Heterozygote	Heterozygote	Heterozygote	Heterozygote
**Patient**	Patient 1	Patient 1	Patient 1	Patient 2	Patient 2	Patient 3	Patient 3
**dbSNP156 rs number**	rs148916596	rs751896015	rs751994566	rs147700251	rs752284064	rs747087320	rs146362213
**1000G**	7.99 × 10^−4^	–	–	2.20 × 10^−3^	–	–	3.59 × 10^−3^
**ExAC**	1.84 × 10^−4^	8.48 × 10^−6^	7.47 × 10^−5^	9.31 × 10^−4^	–	2.03 × 10^−4^	1.70 × 10^−3^
**gnomAD**	2.07 × 10^−4^	4.03 × 10^−6^	7.24 × 10^−5^	9.66 × 10^−4^	4.01 × 10^−5^	2.01 × 10^−4^	1.70 × 10^−3^
**BGI in-house exome database**	5.15 × 10^−4^	–	–	5.15 × 10^−4^	–	–	1.03 × 10^−3^
**Our in-house exome database**	**–**	–	–	–	–	–	–
**HGMD**	**–**	–	–	CM1810297	–	–	–
**ClinVar**	Likely benign	Conflicting classification of pathogenicity	Likely benign	Benign	Uncertain significance	Conflicting classification of pathogenicity	Conflicting classification of pathogenicity
**SIFT (score)**	Damaging (0.008)	Tolerated (0.080)	Damaging (0.000)	Tolerated (0.200)	Damaging (0.003)	Tolerated (0.616)	Damaging (0.035)
**PROVEAN (score)**	Neutral (−1.82)	Deleterious (−3.07)	Deleterious (−5.39)	Deleterious (−4.25)	Neutral (−2.09)	Neutral (−0.45)	Deleterious (−4.41)
**PolyPhen-2 (score)**	Benign (0.002)	Benign (0.033)	Probably damaging (0.688)	Benign (0.141)	Benign (0.018)	Benign (0.001)	Benign (0.070)
**CADD (phred score)**	Pathogenic (22.60)	Benign (10.14)	Pathogenic (23.40)	Pathogenic (23.90)	Benign (11.43)	Benign (4.12)	Pathogenic (18.99)
**Missense3D-DB (structural damage)**	**–**	None	None	–	Damaging (buried/exposed switch)	None	None
**MUpro (ΔΔG)**	Decrease stability (−1.78)	Decrease stability (−0.80)	Decrease stability (−1.62)	Decrease stability (−1.53)	Decrease stability (−1.74)	Decrease stability (−1.32)	Decrease stability (−0.57)
**ACMG-AMP**	VUS (PM2 + PM3 + PP1)	VUS (PM2 + PP1)	VUS (PM2 + PP1 + PP3)	VUS (PM2 + PP3)	VUS (PM2)	VUS (PM2 + PM5 + PP1 + BP4)	VUS (PM2 + PP1 + PP3)

ACMG-AMP, American College of Medical Genetics and Genomics-Association for Molecular Pathology; BP, benign supporting; CADD, Combined Annotation Dependent Depletion; dbSNP156, Single Nucleotide Polymorphism database build 156; *DNAH11*, the dynein axonemal heavy chain 11 gene; ExAC, Exome Aggregation Consortium; gnomAD, Genome Aggregation Database; HGMD, Human Gene Mutation Database; PM, pathogenic moderate; PolyPhen-2, Polymorphism Phenotyping version 2; PP, pathogenic supporting; PROVEAN, Protein Variation Effect Analyzer; rs, Reference SNP; SIFT, Sorting Intolerant from Tolerant; VUS, variant of uncertain significance; 1000G, 1000 Genomes Project.

^a^The LiftOver tool (http://genome.ucsc.edu/cgi-bin/hgLiftOver) was utilized to convert genome coordinates.

### Variant bioinformatics analyses

**Fig 3:**
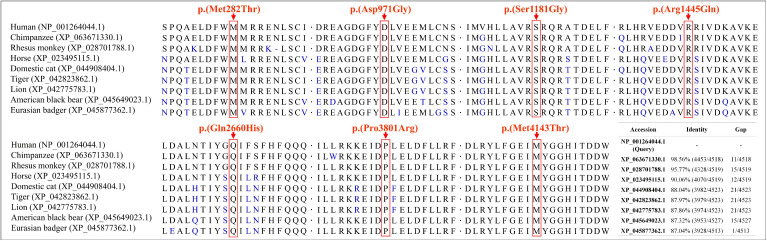
Conservation analysis of the dynein axonemal heavy chain 11 protein, showing that the affected amino acids, indicated by the arrows, are conserved among multiple species.

**Fig 4:**
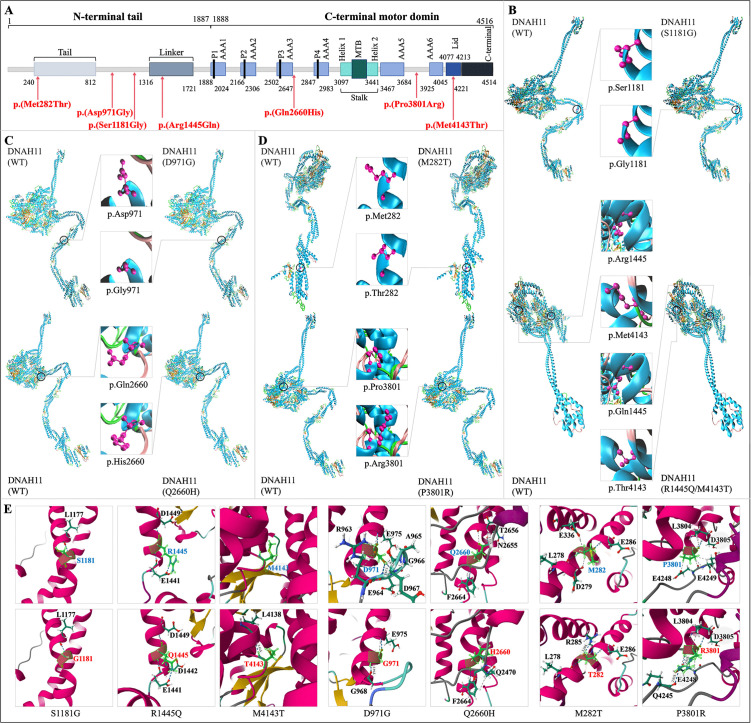
Structural analysis of wild-type and variant-type DNAH11. **(A)** Schematic diagram of the protein with the variants identified in this study indicated. **(B-D)** Cartoon model of the protein visualized by Visual Molecular Dynamics based on the modeling of SWISS-MODEL, and the residue at the mutated position further indicated with ball-and-stick model. **(E)** Non-covalent interactions of residue at the mutated position (represented as ball-and-stick and highlighted in green) calculated by Residue Interaction Network Generator, with the protein part represented as ribbon, interacting residue represented as ball-and-stick model, hydrogen bond shown as blue dotted line, and van der Waals force shown as gray dotted line. AAA, ATPase associated various cellular activities domain; DNAH11, dynein axonemal heavy chain 11; MTB, microtubule-binding domain.

Multi-sequence alignment showed that the affected amino acids were conserved among multiple species (Fig 3). Several online predictive tools showed that these variants may exert deleterious effects and reduce protein stability (Table 2, S1 Table, and S1 Fig). Some variants may affect the critical regions like tail, linker, and lid (Fig 4A). The modeled protein structures were shown in Fig 4B-4D with non-covalent interaction changes induced by the variants depicted in Fig 4E, in which the significant effects on hydrogen bond and van der Waals force were listed in S2 Table. Based on the integration of the hitherto available evidence, the classification of “variant of uncertain significance” (VUS) was proposed following ACMG-AMP’s guidelines.

## Discussion

PCD and LR asymmetry disorder are inherited cilia-related disorders with high clinical and genetic heterogeneity, related to various genes encoding proteins indispensable to ciliary assembly and function. In general, cilia can be divided into two categories including motile cilia and immotile cilia [11]. The motile cilia in the regions like airway, oviduct, and brain ventricles have the “9+2” structure, with two central singlet microtubules surrounded by nine peripheral microtubule doublets, which also exists in the sperm flagellum. With a planar beating, the functional motion involves directional fluid flow and the sperm movement [22]. An exceptional motile cilium, nodal monocilium, only expressed in fetal development and localized to the gastrula ventral node, has the “9+0” structure with dynein, similar to the “9+0” microtubular arrangement of immotile cilium, comprising nine peripheral doublets but lacking the central doublets and radial spokes. With a rotary motion, it generates a leftward fluid flow, which activates the signaling cascade, functioning in LR axis determination in embryogenesis, and the abnormality leads to random organ lateralization [23]. The motor activity of the microtubular dynein complex produces ciliary motion by converting energy into mechanical force and relaying it to the microtubule in an ATP-dependent process. The outer and inner dynein arms (ODAs and IDAs) are attached on the peripheral microtubule doublets, in which the ODAs are comprised of heavy chains, intermediate chains, and light chains [24].

The heavy chain dynein component of the ciliary ODAs, DNAH11, consisting of 4,516 amino acids with a sizable molecular mass of ~520 kDa, is encoded by the *DNAH11* gene (OMIM 603339), located on chromosome 7p15.3 [25]. The protein includes an N-terminal tail and a conserved C-terminal motor domain, which contains six ATPases associated with various cellular activities (AAA1-AAA6), forming a head with AAA+ ring, a stalk with the microtubule-binding domain as its tip, and a lever-like linker close to tail [26]. The β-heavy chain DNAH11 is located in the ciliary proximal region, together with γ-heavy chain DNAH5 in a dimeric form to constitute ODA, which is necessary for ciliary beat production [9,27]. In 2002, the potential involvement of human *DNAH11* gene in PCD was revealed, and a homozygous c.8533C > T (p.(Arg2584*)) variant and a heterozygous c.8990G > A (p.(Arg2997Gln)) variant were reported in two individuals with SI, respectively, which was proposed as one form of PCD [28]. Subsequently, various *DNAH11* gene variants, like c.12363C > G (p.(Tyr4121*)) and c.13531_13587del (p.(Ala4511_Ala4516delinsGln)) in the compound heterozygous form [24], c.4333C > T (p.(Arg1445*)) in the homozygous form, and c.10324C > T (p.(Gln3442*)) in the heterozygous form with an unknown allelic variant were successively reported in patients with PCD, manifesting as respiratory distress, chronic oto-sino-pulmonary disorder, reduced fertility, and random organ lateralization, with abnormal ciliary activity but normal ultrastructure [29]. Using dual-axis electron tomography, PCD-causing *DNAH11* gene variants were further found to result in the loss of proximal ODA volume, once thought to be associated with “normal” ciliary ultrastructure under traditional transmission electron microscopy, probably responsible for ciliary motion deficiencies like immobility, slow beating with reduced bending, and hyperkinetic beating [9]. Currently, the *DNAH11* gene pathogenic variants have been well confirmed to be responsible for the phenotype (OMIM 611884), primary ciliary dyskinesia 7, with or without SI, and a few cases were reported to have infertility [24,30].

The mouse *Dnah11* gene (previously known as left/right-dynein, *lrd*) homozygous missense variant c.6811G > A (p.(Glu2271Lys)) affecting the AAA2 domain was found to be responsible for the laterality randomization in the classical mouse inversum viscerum model (*iv/iv*). Mice homozygous for the *lrd* targeted variant affecting the catalytic first P-loop exhibited randomized LR development with immotile nodal monocilium but normal ciliary ultrastructure. Further study revealed that the *iv/iv* mice manifested other symptoms like rhinitis, sinusitis, and otitis media, features of PCD, and demonstrated immotile tracheal cilia and reduced sperm motility but normal ultrastructure of tracheal cilia and sperm tails, showing an excellent model of PCD [31–33].

Variants in the known PCD genes are responsible for approximately 70% of cases, and more genes remain to be detected. The genetic heterogeneity accounts for the variability in ciliary defects and clinical phenotypes. Combined with the unspecific and time-changing symptoms, limited knowledge of genotype-phenotype correlations warrants probing [6]. Due to no known perfect diagnostic test for PCD, diagnostic guidelines from the European Respiratory Society and American Thoracic Society both recommend combined tests for the diagnosis. By genetic testing, identifying pathogenic variants in the PCD-causing genes can confirm the diagnosis, especially in cases with “normal” traditional transmission electron microscopy findings [34,35]. In this study, we found seven *DNAH11* gene variants in three typical PCD or LR asymmetry disorder patients, consistent with reports that *DNAH11* is the causative gene for PCD, suggesting that these variants may be the genetic cause.

In patient 1, three *DNAH11* variants, c.3541A > G (p.(Ser1181Gly)), c.4334G > A (p.(Arg1445Gln)), and c.12428T > C (p.(Met4143Thr)), were identified. The c.3541A > G (p.(Ser1181Gly)) variant, located in exon 18, results in the substitution of a polar hydrophilic serine with glycine, a small non-polar hydrophobic amino acid lacking a side chain, and disrupts the hydrogen bond between serine (S1181) and leucine (L1177). The c.4334G > A (p.(Arg1445Gln)) variant, located in exon 24, affects a conserved positively charged arginine within a lever-like linker region involved in interactions with the AAA+ ring that are essential for dynein motor activity. The arginine (R1445) was predicted to participate in hydrogen bonding with neighboring acidic residues (E1441 and D1449), and the replacement by glutamine alters these interactions, as well as van der Waals forces, and thereby may affect local linker conformation [36,37]. A nonsense variant c.4333C > T (p.(Arg1445*)) involving the same residue was reported in three PCD families in a compound heterozygous form with c.6203G > A (p.(Arg2068His)), c.2712G > A (p.(Trp904*)), and c.4942C > T (p.(Gln1648*)), respectively, and the homozygous form was also reported in two Caucasian PCD patients, manifesting as neonatal respiratory distress, otitis media, and sinusitis with various situs status (SS/SI), but normal ciliary ultrastructure [29,38,39]. A nearby missense variant c.4457T > A (p.(Leu1486Gln)) involving the same domain, in a compound heterozygous form with c.10006G > T (p.(Ala3336Ser)), was identified in a Chinese boy with PCD, who had respiratory distress, with nearly normal ciliary function, and shared some clinical features with our case, including SS, bronchiectasis, chronic cough, otitis media, sinusitis, and hearing loss [40]. The identified c.12428T > C (p.(Met4143Thr)) variant, located in exon 76, affects the conserved hydrophobic residue within the AAA lid domain associated with ATP binding and protein motion. Replacement of methionine by threonine, introducing a polar side chain, may enable additional hydrogen-bonding interactions with the neighboring leucine (L4138), potentially influencing local conformational flexibility. It was previously reported in a compound heterozygous form with c.9484-1G > T in a Chinese patient with asthenozoospermia, an anomaly reported in approximately 90% of PCD males [41]. Of note, the patient in our report denied infertility, in which the compensatory effect of homologous proteins or clinical heterogeneity may be the underlying mechanism. A nearby missense variant c.12460C > T (p.(Arg4154Cys)) involving the same AAA lid domain, in a compound heterozygous form with c.10877C > A (p.(Pro3626Gln)), was identified in a Pakistani girl with PCD, who had respiratory distress, with abnormal ciliary ultrastructure (ODA and IDA defects), and shared some clinical features with our case, including SS, bronchiectasis, otitis media, and sinusitis [42].

In patient 2, the *DNAH11* variants c.2912A > G (p.(Asp971Gly)) and c.7980A > T (p.(Gln2660His)) were identified. The identified c.2912A > G (p.(Asp971Gly)) variant, located in exon 15, affects the negatively charged acidic hydrophilic residue. Replacement by glycine destroys the hydrogen-bonding interactions with the neighboring acidic residues (E964 and D967), but enables a new hydrogen bond with the nearby glycine (G968), as well as van der Waals forces, which may alter local hydrogen-bonding patterns, potentially influencing regional structural stability. The variant, in a compound heterozygous form with c.11396G > A (p.(Ile3799Thr), was previously detected in a Chinese bronchiectasis sufferer with unreported situs status [43]. The identified c.7980A > T (p.(Gln2660His)) variant, located in exon 49, changes the neutral glutamine to a basic one. Replacement by histidine abolishes the hydrogen-bonding interaction with the neighboring asparagine (N2655) and leads to the residue exchange between buried and exposed state, thereby potentially influencing protein structural stability. These two variants, as a novel combination, may be the disease-causing variants of SI in this patient, which may explain the phenotype difference.

In patient 3, the *DNAH11* variants c.845T > C (p.(Met282Thr)) and c.11402C > G (p.(Pro3801Arg)) were identified. The identified c.845T > C (p.(Met282Thr)) variant, located in exon 4, results in a change from a non-polar hydrophobic to a polar hydrophilic residue within the N-terminal tail. The introduction of a polar side chain enables a new hydrogen-bonding interaction with the nearby arginine (R285), potentially altering local structural properties involved in heavy-chain dimerization and interaction with the ODA intermediate and light chain complex [44]. A missense variant c.846G > C (p.(Met282Ile)) involving the same residue, in a compound heterozygous form with c.2406G > A (p.(Trp802*)), was reported in a Chinese child with nasosinusitis, SA, congenital heart disease, and abnormal ciliary function, sharing the laterality randomization with our reported case [40,45]. A nearby missense variant c.1300T > C (p.(Phe434Leu)), in a compound heterozygous form with c.6983C > T (p.(Pro2328Leu)), was identified in a Chinese bronchiectasis sufferer [45]. The identified c.11402C > G (p.(Pro3801Arg)) variant, located in exon 70, induces a change from the neutral proline to a basic one. Replacement of proline by arginine introduces a flexible positively charged side chain and enables new hydrogen-bonding interactions with the residues (Q4245 and E4248), potentially affecting local structural organization.

Our study identified seven *DNAH11* variants, as a novel combination, in three unrelated PCD or LR asymmetry disorder patients, absent in 876 Chinese controls. The rarity of *DNAH11* variants in our screened cohort, with each variant identified in only one case and absent or present at a low frequency (lower than 3.55 × 10^−4^) in controls, underscores the potential clinical significance. Only the patient 1 presented with SS, bronchiectasis, chronic rhinitis, sinusitis, and otitis media, which is consistent with the existing diagnosis algorithms of PCD. But patient 2 and 3 only exhibited LR asymmetry disorder (SI), a feature of PCD, and it may be regarded as an atypical form of PCD. Due to privacy concerns, detailed family history including chronic respiratory symptoms and autoimmune conditions in relatives could not be obtained. However, available information confirmed absence of consanguinity and comorbidities in the patients. We acknowledge that the lack of extended family clinical data limits genotype-phenotype correlation and interpretation of atypical presentations. With the enrollment of offspring, the *in trans* configuration of two heterozygous variants in patient 1 and 3 can be confirmed. The two heterozygous variants in patient 2 exhibited markedly different allele frequencies across public and in-house databases (e.g., c.2912A > G: 2.20 × 10^−3^ in 1000 Genomes Project, c.7980A > T: absent), making *in cis* inheritance unlikely. However, without parental or offspring samples, compound heterozygosity remains presumptive rather than confirmed. The ACMG-AMP criteria applied “VUS” classification for variants we identified is not uncommon in the PCD-causing genes, which is closely related to the insufficient evidence. Five missense variants we identified were novel, lacking the evidence of pathogenicity in the literature (Table 3). For patient 2, the parents’ refusal made the impracticability of the intrafamilial co-segregation analysis. Functional validation is warranted to confirm variant pathogenicity. However, the large size of the *DNAH11* gene and its encoded protein currently hampers the implementation of functional studies to directly assess the damaging effects of identified variants. With the emergence of improved technology, recurrent and novel variants in more cases with the same disorder can be detected, as well as other supporting evidence. Then, the current “VUS” classification can be reassessed, potentially upgrading to “likely pathogenic” or “pathogenic”, which may be a common scenario as lacking sufficient evidence for rare variants is frequent [46,47]. More functional assays may be achievable via experimental systems like *in vitro* modeling and mimicking animal models, integrating strategies like structural biology and functional genomics, which can further validate variant-disease association and pathogenesis [48,49]. Due to the clinical and genetic overlap of LR asymmetry disorder and PCD, with the development of molecular diagnostics and disease reclassification, these three patients are anticipated to be diagnosed with typical/atypical PCD, or *DNAH11*-associated ciliopathy [6].

**Table 3:** Summary of identified *DNAH11* variants with the same variant sites as our study.

Group^a^	Patient	Ethnicity	Sex	Zygosity	Situs	RDS	Bxsis	Chronic wet cough	Otitis media	Sinusitis	AZS	TEM	CBP	ACMG-AMP	Reference
Ⅱ	#5031	Chinese	M	CH	SA	NA	NA	NA	NA	NA	NA	NA	Immotile, restricted, discordance	c.846G>C (p.(Met282Ile)): LP (PS1 + PM2 + PM3 + PP1)	Liu et al., 2019 [45]
Ⅱ	P44	Chinese	M	CH	SA	No	NA	No	NA	Yes	NA	Normal	Restricted		Guo et al., 2020 [40]
Ⅰ	B142	Chinese	NA	CH	NA	NA	Yes	NA	NA	NA	NA	NA	NA	c.2912A>G (p.(Asp971Gly)): VUS (PM2 + PP3)	Guan et al., 2018 [43]
Ⅱ	PCD1022	Caucasian	M	Hom	SS	Yes	No	NA	Yes	Yes	NA	Normal	NA	c.4333C>T (p.(Arg1445*)): P (PVS1 + PS1 + PM3 + PP1)	Knowles et al., 2012 [29]
Ⅱ	PCD1023	Caucasian	M	Hom	SI	Yes	No	NA	Yes	Yes	NA	Normal	NA		
Ⅱ	HCMJ2	NA	NA	CH	SS	NA	NA	NA	NA	NA	NA	NA	NA		Boon et al., 2014 [38]
Ⅱ	HCMJ101	NA	NA	CH	SS	NA	NA	NA	NA	NA	NA	NA	NA		
Ⅱ	1-28	NA	NA	CH	NA	NA	NA	NA	NA	Yes	NA	Normal	NA		Schramm et al., 2023 [39]
Ⅰ	P1	Chinese	M	CH	NA	NA	NA	NA	NA	NA	Yes	NA	NA	c.12428T>C (p.(Met4143Thr)): VUS (PM2)	Zhu et al., 2019 [41]

ACMG-AMP, American College of Medical Genetics and Genomics-Association for Molecular Pathology; AZS, asthenozoospermia; Bxsis, bronchiectasis; CBP, cilia beat pattern; CH, compound heterozygote; *DNAH11*, the dynein axonemal heavy chain 11 gene; Hom, homozygote; LP, likely pathogenic; M, male; NA, not available; P, pathogenic; PM, pathogenic moderate; PP, pathogenic supporting; PS, pathogenic strong; PVS pathogenic very strong; RDS, respiratory distress; SA, situs ambiguous; SI, situs inversus; SS, situs solitus; TEM, transmission electron microscopy; VUS, variant of uncertain significance.

^a^Group Ⅰ indicates the same variant as we identified in this study, and group Ⅱ indicates variants involving the same codons as we identified.

In summary, our study identified heterozygous *DNAH11* variants, c.[3541A > G];[4334G > A;12428T > C] (p.[(Ser1181Gly)];[(Arg1445Gln;Met4143Thr)]), c.2912A > G(;)7980A > T (p.(Asp971Gly)(;)(Gln2660His)), and c.[845T > C];[11402C > G] (p.[(Met282Thr)];[(Pro3801Arg)]), in three unrelated patients with PCD or LR asymmetry disorder. The various phenotypes including SS, SI, chronic rhinitis, and bronchiectasis indicated that these patients have PCD, even an atypical form of PCD presenting LR asymmetry disorder. The discoveries expand the phenotypic spectrum of *DNAH11* variants and may facilitate more accurate genetic diagnosis and counseling.

## Conclusion

In this study, *DNAH11* disease-associated variants, c.[3541A > G];[4334G > A;12428T > C] (p.[(Ser1181Gly)];[(Arg1445Gln;Met4143Thr)]) in compound heterozygotes (patient 1), c.2912A > G(;)7980A > T (p.(Asp971Gly)(;)(Gln2660His)) in potential compound heterozygotes (patient 2), and c.[845T > C];[11402C > G] (p.[(Met282Thr)];[(Pro3801Arg)]) in compound heterozygotes (patient 3) were identified as potential pathogenic factors responsible for PCD or left-right asymmetry disorder, which could be considered as typical/atypical PCD, or the *DNAH11*-associated ciliopathy.

## Supporting information

S1 Table Information for the selected *DNAH11* benign variants (negative controls) and reported pathogenic variants (positive controls). (PDF)

S1 Fig Comparison of ΔΔG among three groups: negative controls (benign variants), positive controls (reported pathogenic variants), and our variants. ** *P* < 0.01; *** *P* < 0.001; ns, not significant.
(PDF)

S2 Table Changes in hydrogen bonds and van der Waals forces caused by the *DNAH11* gene variants. (PDF)
