# Supplementary material for: Variants in the DNAH11 gene responsible for primary ciliary dyskinesia or probably atypical primary ciliary dyskinesia presenting left-right asymmetry disorder
Source: PLoS One. 2026 May 8;21(5):e0348352. doi: 10.1371/journal.pone.0348352 (PMC13155666; doi:10.1371/journal.pone.0348352)
Supplement: S1 Table — (PDF) [file pone.0348352.s001.pdf]

S1 Table. Information for the selected *DNAH11* benign variants (negative controls) and reported pathogenic variants (positive controls).

Variant No.	Variant	Allele frequency[a]	ClinVar	HGMD	MUPro (ΔΔG)
**NC1**	p.Glu34Val	4.81×10^{-1}	Benign	-	Decrease stability (-0.11)
**NC2**	p.Thr400Ile	4.48×10^{-2}	Benign/Likely benign	-	Decrease stability (-0.11)
**NC3**	p.Thr1038Ala	8.19×10^{-1}	Benign	-	Decrease stability (-0.42)
**NC4**	p.Val2518Ile	3.58×10^{-2}	Benign	-	Decrease stability (-0.33)
**NC5**	p.Val3708Leu	6.40×10^{-1}	Benign	-	Increase stability (0.24)
**NC6**	p.Thr4170Ile	4.12×10^{-1}	Benign/Likely benign	-	Decrease stability (-0.11)
**NC7**	p.Lys4501Arg	7.60×10^{-2}	Benign/Likely benign	-	Decrease stability (-0.34)
**PC1**	p.Ala382Gly	-	-	CM206909	Decrease stability (-1.16)
**PC2**	p.Phe434Leu	-	-	CM1810305	Decrease stability (-1.45)
**PC3**	p.Trp1222Arg	-	-	BM1498194	Decrease stability (-1.05)
**PC4**	p.Leu1486Gln	-	-	-	Decrease stability (-2.02)
**PC5**	p.Arg1834Gly	1.24×10^{-6}	Uncertain significance	CM140771	Decrease stability (-1.83)
**PC6**	p.Ala4022Pro	-	Likely pathogenic	CM1110372	Decrease stability (-0.80)
**PC7**	p.Arg4154Cys	6.82×10^{-6}	Uncertain significance	CM148016	Decrease stability (-0.49)

DNAH11, the dynein axonemal heavy chain 11 gene; HGMD, Human Gene Mutation Database; NC, negative control; PC, positive control.

[a]Allele frequency from Genome Aggregation Database.
