# Supplementary material for: Variants in the DNAH11 gene responsible for primary ciliary dyskinesia or probably atypical primary ciliary dyskinesia presenting left-right asymmetry disorder
Source: PLoS One. 2026 May 8;21(5):e0348352. doi: 10.1371/journal.pone.0348352 (PMC13155666; doi:10.1371/journal.pone.0348352)

S1 Fig. Comparison of ΔΔG among three groups: negative controls (benign variants), positive controls (reported pathogenic variants), and our variants. ** $P < 0.01$; *** $P < 0.001$; ns, not significant.

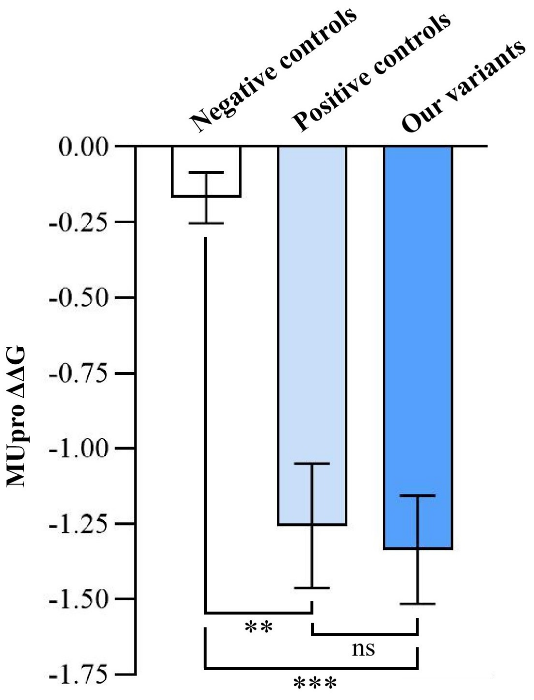

Supplement: S1 Fig — ** P < 0.01; *** P < 0.001; ns, not significant. (PDF) [file pone.0348352.s002.pdf]
