# Supplementary material for: Variants in the DNAH11 gene responsible for primary ciliary dyskinesia or probably atypical primary ciliary dyskinesia presenting left-right asymmetry disorder
Source: PLoS One. 2026 May 8;21(5):e0348352. doi: 10.1371/journal.pone.0348352 (PMC13155666; doi:10.1371/journal.pone.0348352)
Supplement: S2 Table — (PDF) [file pone.0348352.s003.pdf]

S2 Table. Changes in hydrogen bonds and van der Waals forces caused by the *DNAH11* gene variants.

Variant type	ResPos[a]	Hydrogen bond	Van der Waals force
**Wild 1**	S1181	L1177 (Distance: 2.85 Å; Angle: 163.02°)	-
**Variant 1**	G1181	L1177 (Distance: 2.84 Å; Angle: 160.37°)	-
**Wild 2**	R1445	E1441 (Distance: 2.87 Å; Angle: 161.48°)<br>E1441 (Distance: 2.63 Å; Angle: 160.56°)<br>D1449 (Distance: 2.83 Å; Angle: 157.44°)	-
**Variant 2**	Q1445	E1441 (Distance: 2.90 Å; Angle: 123.72°)<br>E1441 (Distance: 2.72 Å; Angle: 165.15°)<br>D1449 (Distance: 2.73 Å; Angle: 161.91°)	**E1441 (Distance: 2.90 Å)**<br>**E1441 (Distance: 3.25 Å)**<br>**D1442 (Distance: 2.69 Å)**<br>**D1449 (Distance: 2.92 Å)**
**Wild 3**	M4143	-	-
**Variant 3**	T4143	**L4138 (Distance: 3.41 Å; Angle: 158.10°)**	-
**Wild 4**	D971	**E964 (Distance: 3.19 Å; Angle: 138.46°)**<br>**D967 (Distance: 2.87 Å; Angle: 149.76°)**<br>E975 (Distance: 2.75 Å; Angle: 164.12°)	**R963 (Distance: 2.73 Å)**<br>**A965 (Distance: 3.23 Å)**<br>**G966 (Distance: 2.69 Å)**<br>**D967 (Distance: 2.90 Å)**<br>E975 (Distance: 2.93 Å)
**Variant 4**	G971	**G968 (Distance: 2.91 Å; Angle: 124.08°)**<br>E975 (Distance: 2.79 Å; Angle: 159.57°)	E975 (Distance: 2.90 Å)
**Wild 5**	Q2660	**N2655 (Distance: 3.21 Å; Angle: 136.01°)**<br>F2664 (Distance: 3.21 Å; Angle: 169.60°)	**T2656 (Distance: 3.06 Å)**<br>**F2664 (Distance: 2.74 Å)**
**Variant 5**	H2660	F2664 (Distance: 3.38 Å; Angle: 170.65°)	**Q2470 (Distance: 3.26 Å)**<br>**Q2470 (Distance: 3.19 Å)**
**Wild 6**	M282	L278 (Distance: 2.98 Å; Angle: 169.57°)<br>E286 (Distance: 2.94 Å; Angle: 159.67°)	**D279 (Distance: 2.70 Å)**<br>**E336 (Distance: 2.91 Å)**
**Variant 6**	T282	L278 (Distance: 3.08 Å; Angle: 171.78°)<br>**R285 (Distance: 3.20 Å; Angle: 144.96°)**<br>E286 (Distance: 2.92 Å; Angle: 161.44°)	-
**Wild 7**	P3801	D3805 (Distance: 2.84 Å; Angle: 162.35°)	L3804 (Distance: 2.89 Å)<br>L3804 (Distance: 2.71 Å)<br>**D3805 (Distance: 2.92 Å)**<br>E4248 (Distance: 3.23 Å)<br>E4248 (Distance: 2.89 Å)<br>**E4249 (Distance: 2.75 Å)**<br>**E4249 (Distance: 2.88 Å)**
**Variant 7**	R3801	D3805 (Distance: 2.90 Å; Angle: 161.05°)<br>**Q4245 (Distance: 2.82 Å; Angle: 134.95°)**<br>**E4248 (Distance: 2.83 Å; Angle: 159.28°)**	L3804 (Distance: 2.91 Å)<br>L3804 (Distance: 2.89 Å)<br>E4248 (Distance: 2.90 Å)<br>E4248 (Distance: 2.73 Å)

DNAH11, the dynein axonemal heavy chain 11 gene.

[a]ResPos, the residue at the mutated position.
